# Supplementary material for: Multifunctional Three-Dimensional Printed Copper Loaded Calcium Phosphate Scaffolds for Bone Regeneration
Source: Pharmaceuticals (Basel). 2023 Feb 25;16(3):352. doi: 10.3390/ph16030352 (PMC10052742; doi:10.3390/ph16030352)
Supplement: Supplementary file 1 [file pharmaceuticals-16-00352-s001.zip › pharmaceuticals-2165067-supplementary.pdf]

Supplementary Information

Multifunctional three-dimensional printed copper loaded calcium phosphate scaffolds for bone regeneration

Amit Pillai[#], Jaidev Chakka[#], Niloofar Heshmati, Yu Zhang, Faez Alkadi, and Mohammed Maniruzzaman*

PharmE3D lab, Division of Molecular Pharmaceutics, College of Pharmacy, The University of Texas at Austin, TX 78712, USA

*Corresponding Author; [#]Equal contribution

Results

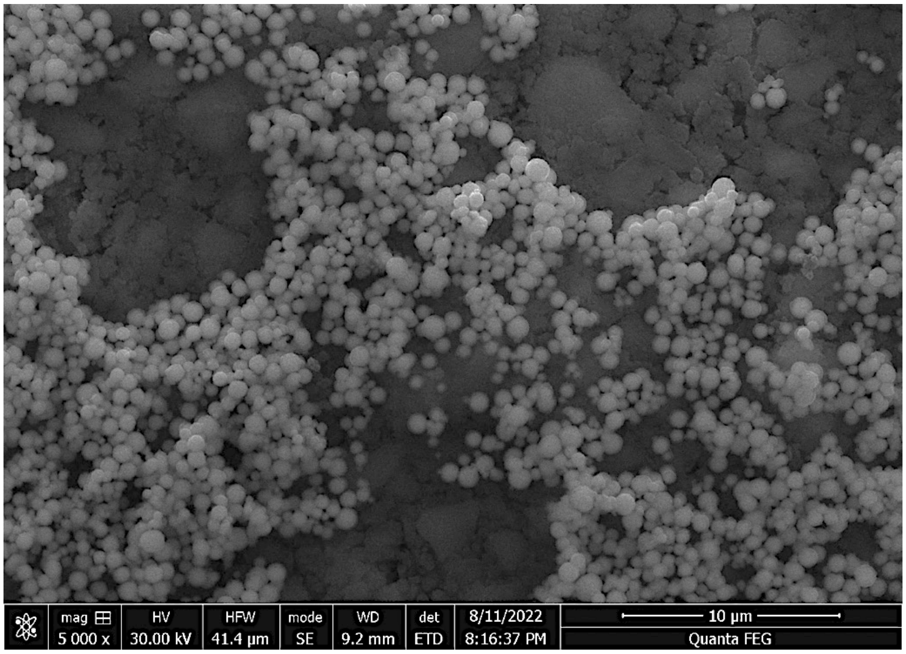

Figure S1: The scanning electron microscopy of copper nanoparticles with an average particle size of 300 nm procured from a commercial vendor.
